# Commentary: Integrating electrodermal biofeedback into pharmacologic treatment of grand mal seizures

**DOI:** 10.3389/fnhum.2015.00666

**Published:** 2015-12-23

**Authors:** Iliana Kotwas, Jean-Arthur Micoulaud-Franchi, Fabrice Bartolomei, Yoko Nagai

**Affiliations:** ^1^Laboratoire Parole et Langage (UMR 7309), Aix-Marseille UniversitéMarseille, France; ^2^Service d'Explorations Fonctionnelles du Système Nerveux, Clinique du Sommeil, Centre Hospitalier Universitaire de BordeauxBordeaux, France; ^3^Centre National de la Recherche Scientifique, USR 3413 SANPSY, Centre Hospitalier Universitaire Pellegrin, Université de BordeauxBordeaux, France; ^4^Service de Neurophysiologie Clinique, Centre Hospitalier Universitaire de la TimoneMarseille, France; ^5^Institut National de la Santé et de la Recherche Médicale, UMR 751 Epilepsie et CognitionMarseille, France; ^6^Psychiatry, Brighton and Sussex Medical School, University of SussexLondon, UK; ^7^Department of Clinical and Experimental Epilepsy, Institute of Neurology, University College LondonLondon, UK

**Keywords:** biofeedback, electrodermal activity, sympathetic arousal, epilepsy, self-efficacy

Thirty percent of patients with epilepsy experience seizures despite optimal anticonvulsant drug treatment. Stress is frequently identified by patients with epilepsy as a precipitant of seizures (Spector et al., 2000; Ferlisi and Shorvon, 2014). Patients also often report using countermeasures to control the seizure onset. These are typically spontaneous and individualized such as increasing arousal by walking, breathing, standing, focusing attention, changing way of thinking, and more rarely inducing relaxation (Lee and No, 2005; Hether et al., 2013). In parallel, behavioral and psychological interventions, complementing conventional therapeutic methods for the management of epileptic seizures, have gained greater clinical attention over the past decade. Among these, Biofeedback (BFK) represents a noninvasive biobehavioral treatment that enables a patient to gain volitional control over a specific physiological process. BFK has already shown its value when applied to patients with epilepsy (Sterman and Friar, 1972; Rockstroh et al., 1993; Nagai et al., 2004a; Nagai, 2011; Micoulaud-Franchi et al., 2014a,b). Scrimali et al. (2015) have rightly pointed out the potential usefulness of electrodermal biofeedback in the management of refractory epilepsy. In a single case study, they report an effect of electrodermal activity (EDA) relaxation biofeedback in reducing seizures in a patient treated for 2 years. This case study supports the necessity to expand clinical armamentarium for treatment-resistant patients with few alternatives.

However, in this commentary we would like to specify that contrary to what it is claimed by the authors, decreasing arousal, using relaxation biofeedback is not the method proposed by Nagai et al. (2004a). On the contrary, these authors used a protocol based on increasing arousal. The EDA biofeedback treatment designed by Nagai et al. (2004a) is aimed at increasing the tonic levels of peripheral sympathetic arousal rather than at decreasing EDA level. In addition, these authors (Scrimali et al., 2015) cite Nagai et al. (2004a) when they claim that “Grand Mal” seizures are characterized by increased sympathetic arousal. However, there is no mention of such a relationship in the paper of Nagai et al. that rather highlights an inverse relationship between sympathetic arousal and an electroencephalographic index of cortical arousal that is linked with seizure activity.

The motivation for believing that EDA relaxation biofeedback may be beneficial for patients with epilepsy comes is understandable given the relationship between stress and increased skin conductance level. It is known that stress and anxiety are commonly reported triggers of seizures (Fenwick, 1991; Antebi and Bird, 1993; Spector et al., 2001; Nakken et al., 2005; Petitmengin et al., 2006; Pinikahana and Dono, 2009). Thus, inducing relaxation in order to reduce the impact of stress has theoretical justification for patients with epileptic seizure triggered by stress. EDA reflects the action of sympathetic autonomic nerves on eccrine sweat glands and is a sensitive indicator of central (involuntary and voluntary induced) changes in arousal, associated with emotion, attention, and physical activity (Neumann and Blanton, 1970). In fact, EDA can be increased not only by stress, but also by preparation of and engagement with intellectual activity, during emotion regulation or even when experiencing happiness (Gross and Levenson, 1993; Reynaud et al., 2012). Moreover, regarding the relationship between autonomic physiology and seizures in epilepsy, a state of enhanced relaxation/low arousal can potentially worsen seizures through lowering the seizure threshold (Nagai et al., 2004b). The theoretical rational behind the development of EDA biofeedback originated with a neurofeedback protocol focusing on Slow Cortical Potentials (SCP), an index of thalamocortical excitability. In SCP neurofeedback, patients are trained to reduce and reverse the negative amplitude of SCPs (Kotchoubey et al., 2001). This neurofeedback method has been shown to elicit a significant decrease in seizure frequency (Rockstroh et al., 1993) and can produce a sustained benefit that is apparent ten years after the end of treatment (Strehl et al., 2014). The amplitude of the Contingent Negative Variation (CNV) (a task-evoked event-related potential considered as a SCP and hence an index of cortical excitation) is greater during states of reduced autonomic arousal (Nagai et al., 2004b, 2009). Peripheral sympathetic activity (autonomic arousal), measured using EDA, can be increased either involuntarily by aversive auditory stimulation, or voluntarily by using biofeedback arousal. In both cases, the increase is associated with a reduction in the amplitude of the CNV. This observation indicated that the measures of peripheral sympathetic activity and thalamocortical excitation (as measured by the CNV) are inversely related (Nagai et al., 2004b). This inverse relationship led to the theoretical proposal that enhancing autonomic arousal may have a therapeutic use in preventing or curtailing the onset of seizures.

Despite strong theoretical underpinnings there is little direct knowledge about the neural mechanisms underlying EDA biofeedback on seizure onset. Scrimali et al. (2015) suggest that Nagai (2014) report that “EDA-biofeedback can improve thalamocortical regulation of neural excitability across brain networks as it focuses on the reduction of peripheral sympathetic tone.” This reference to a Japanese article is inaccurate and confuses the likely manner in which way the regulation occurs. The thalamus certainly has an important role in generation of the CNV (Elbert and Rockstroh, 1987). In a neuroimaging study, CNV-related central neural brain activation was observed to extend beyond thalamus and to engage midline cortices, notably the supplementary motor area (SMA) and mid cingulate (Nagai et al., 2004c). The magnitude of activity within these regions correlated with CNV amplitude on a trial-by-trial basis, suggesting these areas are involved in the generation of CNV. Importantly, a set of related (but distinct) cortical regions (including orbitofrontal and ventral prefrontal cortex, anterior cingulate cortex, insula, and right parietal cortex) are involved in the regulation of EDA and have direct projections to thalamus (Critchley et al., 2000). Moreover, modulation of EDA level using biofeedback exerts a specific regulatory effect on orbital and ventral prefrontal activity (Nagai et al., 2004d). The implication is that EDA arousal biofeedback may thus modulate thalamocortical information flow and consequently enable the volitional reducing seizures occurrence (Nagai, 2011).

As evidence of the clinical efficacy of EDA arousal biofeedback in the management of epileptic seizures, Nagai et al. (2004a) report a mean reduction of seizures of about 50% in a biofeedback treatment group, compared to no difference in seizure frequency in a control group undergoing sham biofeedback. Moreover, across the treatment group, there was a positive correlation between the patients' reduction in seizure frequency and the degree of improvement observed over training sessions in their performance of the biofeedback task. Similar results, notably an improvement in psychometric measures of symptoms of depression and negative affect, are reported in patients with stress-triggered seizures (Micoulaud-Franchi et al., 2014b). This suggests that EDA arousal biofeedback training is applicable to stress-related epilepsy, though perhaps counterintuitive given typical relationship between stress and increased sympathetic arousal. In both cases, patients were taught to target the use of strategies developed during the biofeedback training session to target preictal and ictal periods when increasing arousal can serve as a countermeasure to decrease the risk of seizure onset. The study of Scrimali et al. raises the issue that patients who report stress as main trigger factor, typically identify increases in chronic stress, rather than acute stressors, as seizure precipitants (Privitera et al., 2014). In this way, decreasing stress-related autonomic arousal by relaxation in the interictal period might be specifically helpful for chronic stress management and correspondingly reduce the latent risk of seizures. In this way, the Scrimali et al. report could represent an interesting alternative approach for the use of biofeedback in epilepsy that must be demonstrated in a larger cohort.

In conclusion, EDA biofeedback has great potential as a non-pharmacological therapy for the management of refractory epilepsy. The methodology is grounded on theory, and on increasing knowledge regarding the underlying neural mechanisms, and the approach deserves greater clinical attention. There also remains a need for continuing clinical research, such as the case study report by Scrimali et al. (2015). Despite some inaccurate interpretations of findings of published EDA biofeedback papers, the study of Scrimali et al. rises the need to develop noninvasive biobehavioral treatment that target specifically pré-ictal/ictal periods or interical periods to manage efficiently patients with refractory epilepsy. Further studies that will establish and consolidate the role of EDA biofeedback as a complementary therapy for epilepsy should bring: (i) large well-controlled, randomized, double-blind, and long-term evaluations studies, (ii) optimal implementation and embedding of the training with the EDA biofeedback sessions, (iii) the possibility of incorporation of different forms of bioefeedback and neurofeedback. All these different areas need to be underpinned by neurophysiological rationales.

## Conflict of interest statement

The authors declare that the research was conducted in the absence of any commercial or financial relationships that could be construed as a potential conflict of interest.
